# The role of nerve microenvironment for neurofibroma development

**DOI:** 10.18632/oncotarget.11133

**Published:** 2016-08-09

**Authors:** Chung-Ping Liao, Sanjay Pradhan, Zhiguo Chen, Amish J. Patel, Reid C. Booker, Lu Q. Le

**Affiliations:** ^1^ Department of Dermatology, University of Texas Southwestern Medical Center, Dallas, TX 75390, USA; ^2^ Simmons Comprehensive Cancer Center, University of Texas Southwestern Medical Center, Dallas, TX 75390, USA; ^3^ UTSW Neurofibromatosis Clinic, University of Texas Southwestern Medical Center, Dallas, TX 75390, USA

**Keywords:** neurofibromatosis, neurofibroma, tumor microenvironment, peripheral nerve sheath tumor, NF1

## Abstract

Deregulation of RAS signaling in Neurofibromatosis type 1 (NF1) results in the development of multiple neurofibromas, complex tumor of the peripheral nerves with no effective medical treatment. There is increasing evidences that neurofibroma initiates through loss of *NF1* function in the Schwann cell lineage, followed by a cascade of interactions with other cell types in the surrounding tumor microenvironment. In NF1 patients, neurofibromas always develop along peripheral nerves and do not migrate to distant organs, including the central nervous system. In this study, we sought to identify the contributions of these peripheral nerves in neurofibroma formation. Using *in vivo* and *in vitro* three-dimensional (3D) culturing system, we show that peripheral nerves are absolutely required for neurofibroma tumorigenesis and report a novel 3D skin raft culture system for neurofibroma formation *in vitro* to decipher tumor pathogenesis. This interaction between neoplastic Schwann cells and their surrounding neural microenvironment has important implications for understanding early cellular events that dictate tumorigenesis. It also provides fertile ground for the elucidation of intrinsic and extrinsic factors within the nerve microenvironment that likely play essential roles in neurofibroma development and, therefore, viable therapeutic targets in neurofibroma therapy.

## INTRODUCTION

The tumor predisposition von Recklinghausen's Neurofibromatosis type 1 (NF1) is an autosomal dominant inheritable disorder affecting about 1 in 3000 worldwide, regardless of ethnic origin or gender. NF1 patients have a spectrum of clinical presentations including neurofibromas, cafe-au-lait spots, freckling in axillary or inguinal regions, Lisch nodules, bone deformities, learning disability, myeloid malignancy, glioma, and pheochromocytoma [1, 2].

Neurofibroma is the hallmark manifestation of human NF1. It is a mixed-cell tumor, including the neoplastic Schwann cells, that develops along peripheral nerve sheath. Neurofibroma growing along cutaneous nerve twigs is termed dermal neurofibroma, which is one of the most common features of NF1 and affects the majority of NF1 patients. It appears as numerous soft nodules on the skin and can cause severe disfiguring. The first appearance of dermal neurofibromas in NF1 patients is often around puberty; therefore, the initiation of dermal neurofibroma formation has been suggested to be associated with hormone regulation [3]. On the other hand, plexiform neurofibromas develop along internal nerve plexus and continue to grow throughout life. The prevalence for NF1 patients to develop plexiform neurofibromas is around 30% and they have a 10% lifetime of transforming into the highly malignant soft tissue sarcoma named malignant peripheral nerve sheath tumor (MPNST) [1]. Genome-wide analysis has revealed that MPNSTs harbor additional genetic mutations, such as *P53*, *CDKN2A*, or *SUZ12* in addition to *NF1* mutations [4]. Currently there is no effective medical treatment for both neurofibroma and MPNST, although recent animal studies have suggested that MEK/ERK pathway could be a target for NF1-associated neoplasms, including plexiform neurofibroma but not MPNST [5–7]. This reflects the lack of complete understanding of the biology and pathogenesis of these NF1-associated tumors, including the roles of different cell types within the surrounding tumor microenvironment [8].

The proliferation of many neoplastic cells is sustained by complex cellular and non-cellular components in their tumor microenvironment. In neurofibromas, as mixed-cell tumors, in addition to the neoplastic Schwann cells, the most common cells types associated with this tumor are fibroblasts, endothelial cells, neurons and various types of immune cells including mast cells and macrophages [9]. There is also extracellular matrix, mainly produced by fibroblasts, to support the tumor tissues and to communicate intracellular signalings. Many preclinical experiments and clinical trials have demonstrated that tumor microenvironment is a viable therapeutic target in numerous types of neoplasms, including neurofibromas [10, 11].

Clinically, plexiform neurofibromas always develop along peripheral nerves and do not migrate to distinct organs, including central nervous system. For example, para-spinal plexiform neurofibromas, which develop in or around dorsal root ganglion (DRG), are always restricted in the peripheral nerves even though they are anatomically adjacent to the spinal cord. This phenomenon is fully recapitulated by our genetically engineered mouse models of plexiform neurofibroma [12, 13]. In addition, successful human plexiform neurofibroma mouse xenograft model is limited to orthotopic transplantation in peripheral nerve tissues [14]. Furthermore, primary dermal and plexiform neurofibroma tumor cells isolated from patients have a very limited proliferative capacity *in vitro* and undergo senescence within a short number of passages [15, 16], suggesting the missing of critical prosurvival factors. These observations lead to the hypothesis that peripheral nerves play a critical role in supporting neurofibroma development.

## RESULTS AND DISCUSSION

### *Nf1^−/−^* SKPs give rise to classic neurofibroma in sciatic nerve but not in subcutaneous tissue

We recently identified the population of neural crest-related progenitors residing in the dermis termed skin-derived precursors (SKPs) as the cell of origin for NF1-associated dermal neurofibroma. We show that *Nf1*-deficient SKPs can give rise to classic plexiform or dermal neurofibromas contingent on their local microenvironment and exhibit the same properties as the embryonic Schwann cell progenitors that give rise to plexiform neurofibromas [3, 13, 17–19]. In this study, we employed this model to test the hypothesis that neurofibroma development is dependent on peripheral nerve in its tumor microenvironment. This approach was performed by comparing the tumorigenic activity of *Nf1^−/−^* SKPs in nerve (sciatic nerve) and non-nerve (subcutaneous) tissues in athymic nude mice, which are permissive hosts for SKPs to grow in allograft transplantations.

**Figure 1:**
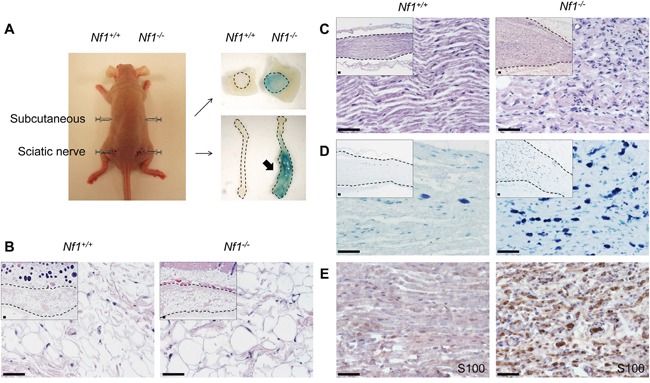
*Nf1*^−/−^ SKPs give rise to classic neurofibroma in sciatic nerve but not in subcutaneous tissue *Nf1^−/−^* SKPs were generated from *Nf1^f/f^;R26R* SKPs followed by Cre-expressing adenovirus transduction. The R26-LacZ reporter was activated by Cre in *Nf1^−/−^* SKPs, resulting in blue color after X-gal staining. *Nf1^+/+^* and *Nf1^−/−^* SKPs were injected under skin and into sciatic nerve. The tissue developed from injected SKPs were harvested after 5 months followed by X-gal staining **A.** In subcutaneous tissue, both *Nf1^+/+^* and *Nf1^−/−^* SKPs developed into tissues within adipocytes **B.** *Nf1^−/−^* but not *Nf1^+/+^* SKPs developed into neurofibroma in sciatic nerve as characterized by H&E staining **C.**, toluidine blue staining for mast cells **D.**, and S100 IHC staining for Schwann cells **E.** Sciatic nerve allografted with *Nf1^+/+^* SKPs injection showed normal nerve structure (C-E). Scale bar = 50 μm.

Our results revealed that neither *Nf1^+/+^* nor *Nf1^−/−^* SKPs can give rise to tumor subcutaneously (Figure 1A and 1B). However, *Nf1^−/−^* SKPs but not *Nf1^+/+^* controls robustly give rise to tumors in sciatic nerves (Figure 1A and 1C). X-gal staining of the tumors demonstrates that the tumor cells were LacZ positive, pointing to their origin from the transplanted SKPs and not from paracrine tumor induction of host sciatic nerve cells. The tumor derived from *Nf1^−/−^* SKPs showed hyperplasia of disorganized cells with wavy nuclei (Figure 1C). To further characterize this tumor, we performed IHC staining for Schwann cell marker S100 and mast cell staining by toluidine blue. Our results showed that the tumor derived from *Nf1^−/−^* SKPs is mainly composed of Schwann cell (Figure 1E) and infiltrated with mast cells (Figure 1D). Taken together, all the histological and molecular characteristics of the tumors derived from *Nf1^−/−^* SKPs in sciatic nerve consistent with those of neurofibromas. In contrast, we did not see any signs of neoplasm in the sciatic nerves implanted with *Nf1^+/+^* SKPs, demonstrating that loss of *Nf1* in SKPs is required but not sufficient to induce tumor formation. Importantly, our discovery that *Nf1^−/−^* SKPs give rise to neurofibroma in sciatic nerves but not subcutaneous tissue indicates an essential role of nerve microenvironment for neurofibroma development. In patients with NF1, trauma has been suggested as one of the associated factors with neurofibroma formation [20]. In fact, nerve injury can corporate with the loss of NF1 to promote tumor development [21, 22]. In this setting, injection of cells into sciatic nerves can cause nerve damage and inflammation, inducing a physiological process of Wallerian degeneration that leads to Schwann cell proliferation. Albeit these nerve injury specific processes or the nerve itself contribute to neurofibroma growth from SKPs is not known at this time, it underscores the critical role of nerve microenvironment for neurofibroma tumorigenesis.

### Nerve microenvironment also promotes MPNST development

**Figure 2:**
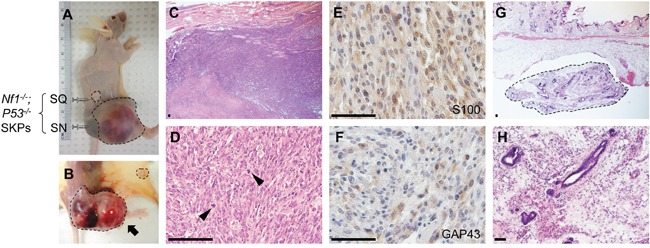
Nerve microenvironment also promotes MPNST development *Nf1^−/−^;P53*^−/−^ SKPs were injected under skin and into sciatic nerve in athymic nude mice to examine the contribution of nerve microenvironment to MPNST development. Tissues developed from *Nf1^−/−^;P53*^−/−^ SKPs were harvested 2 months after injection. *Nf1^−/−^;P53*^−/−^ SKPs proliferate remarkably fast in sciatic nerve but not under skin **A&B.** *Nf1^−/−^;P53*^−/−^ SKPs in sciatic nerve differentiated into malignant tumor **C&D.**, with mitotic figure (arrowhead) and positive staining for S100 **E.** and GAP43 **F.**, consistent with MPNST. *Nf1^−/−^;P53*^−/−^ SKPs differentiate into a small tissue under skin **G&H.** Arrows indicated the tumors formed in sciatic nerve. SQ = subcutaneous, SN = sciatic nerve. Scale bar = 50 μm.

Although plexiform neurofibroma is benign tumor, it possesses the potential to transform into malignant sarcoma MPNST upon harboring additional genetic changes, such as loss of *P53*. Therefore, we sought to determine whether peripheral nerves also play a supportive role for MPNST development. By using the same strategy, we compared the tumorigenic activity of *Nf1^−/−^;P53*^−/−^ SKPs implanted in sciatic nerves and subcutaneous tissue in nude mice. Our results showed that *Nf1^−/−^;P53*^−/−^ SKPs proliferate remarkably faster in sciatic nerves than in subcutaneous tissue (Figure 2A and 2B). In sciatic nerve, a palpable mass was formed in less one month and it rapidly grew to 2-cm in 2 months (Figure 2A and 2B). Histological analysis revealed that these tumors in sciatic nerves showed hyperplastic of spindle cells, abundant mitotic figures, pleomorphic nuclei, and high nuclear-cytoplasmic ratio (Figure 2C and 2D), and positive staining of Schwann cell markers S100 and GAP43 (Figure 2E and 2F); all of these features match MPNST characteristics. Under the same time setting, the implanted cells in subcutaneous tissue did not from malignant tumor (Figure 2G and 2H). Our findings that *Nf1^−/−^;P53*^−/−^ SKPs rapidly gave rise MPNST in nerve tissue demonstrates that nerve microenvironment is crucial for MPNST development. It has been shown that the transformation into MPNST can be driven by Nrg1 [23], a well characterized neuronal factor that dictates Schwann cell proliferation and differentiation [24]. This is consistent with our findings that nerve microenvironment also plays an important role in promoting MPNST tumorigenesis.

### *Nf1*^−/−^ SKPs give rise to neurofibroma in subcutaneous tissue with nerve reconstitution

**Figure 3:**
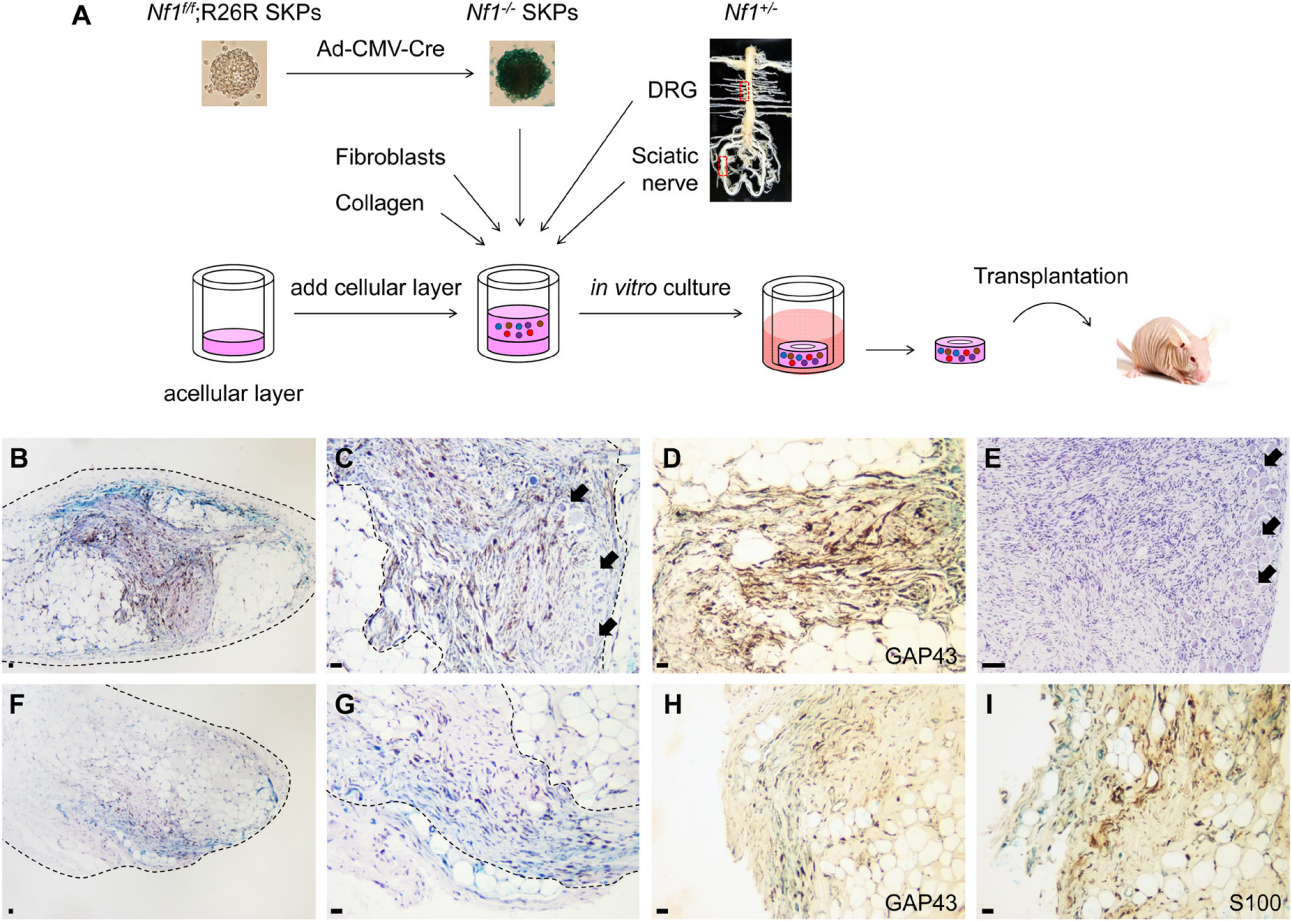
*Nf1*^−/−^ SKPs give rise to neurofibroma in subcutaneous tissue with nerve reconstitution Schematic representation of the experimental design **A.** *Nf1^−/−^* SKPs were co-cultured with *Nf1^+/−^* DRGs **B-D.** or *Nf1^+/−^* sciatic nerves **F-I.** in an *in vitro* raft culture for 5 days followed by transplantation into athymic nude mice subcutaneously. The rafts were harvested 5 months after transplantation followed by X-gal staining. H&E staining revealed that *Nf1^−/−^* SKPs differentiated into neurofibroma in grafted raft with either DRGs (B&C) or sciatic nerves (F&G). Spontaneous para-spinal plexiform neurofibroma developed in *Nf1^f/−^;PLPCre^ERT2^+* mice with 4OHT induction at neonatal stage was shown as control **E.** IHC staining for GAP43 (D&H) and S100 (I) confirmed that *Nf1^−/−^* SKPs differentiated into Schwann cells in both neurofibromas. Arrows indicated the DRG neuron bodies. Scale bar = 50 μm.

To confirm the peripheral nerve dependence during neurofibroma development, we explored the tumorigenic activity of *Nf1^−/−^* SKPs in subcutaneous tissue with nerve reconstitution. In this approach, we first developed an *in vitro* engineered 3D skin raft culture with *Nf1^−/−^* SKPs and *Nf1^+/−^* DRGs which represent nerve tissues containing peripheral neuron bodies. These skin rafts were constructed with layers of collagen type I and human dermal fibroblasts as skin structure [25–27], in addition to *Nf1^−/−^* SKPs as tumor-initiating cells for neurofibroma, and *Nf1^+/−^* nerve tissues as environmental factors. These skin rafts were initially maintained in *in vitro* culture for 5 days and then transplanted into the skin of nude mice *in vivo* (Figure 3A). Strikingly, 5 months after transplantation, we observed that *Nf1^−/−^* SKPs gave rise to tumor with disorganized hypercellularity. These hyperplastic cells were stained positive for LacZ, demonstrating the cellular origin from *Nf1^−/−^* SKPs (Figure 3B). These cells also showed neurotropic pattern by clustering with DRG neuron bodies (Figure 3C). Furthermore, immunostaining of GAP43 identified them as Schwann cells (Figure 3D), consistent with the fundamental signatures of neurofibroma. We further compared this neoplasm with para-spinal plexiform neurofibroma originated from *Nf1^flox/−^;PLPCre^ERT2^+* mice [12] and found they indeed shared a high similarity of histological and molecular featured of disorganized hyperplastic spindle-shaped Schwann cells surrounding DRG neuron bodies (Figure 3C and 3E). In a similar experiment, we replaced *Nf1^+/−^* DRGs with *Nf1^+/−^* sciatic nerves which contain peripheral neuron axon fibers to further characterize this nerve dependency. Strikingly, in this case, *Nf1^−/−^* SKPs also gave rise to classic neurofibroma (Figure 3F-3I). Taken together, our novel skin raft allograft with nerve reconstitution assays revealed that nerve tissues containing either peripheral neuron bodies or axon fibers are sufficient to support neurofibroma development. The DRG and sciatic nerve tissues are composed of peripheral neural cells (neurons and Schwann cells) as well as other cellular (eg. fibroblasts and endothelial cells) and non-cellular (eg. extracellular matrix) elements. Given the fact that *Nf1^−/−^* SKPs cannot give rise to neurofibroma in subcutaneous tissue that contains all of these non-neural components, the critical factor which drives *Nf1^−/−^* SKPs to undergo tumorigenesis is likely to be peripheral neurons.

### Three-dimensional skin raft culture system for neurofibroma formation *in vitro*

**Figure 4:**
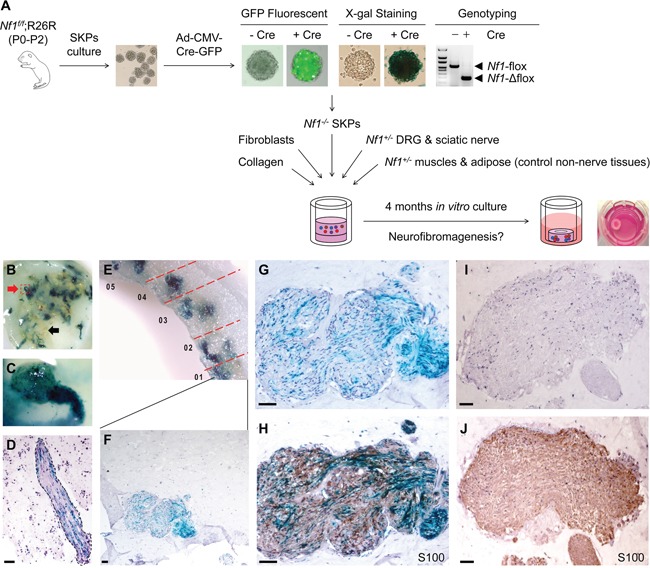
Three-dimensional skin raft culture system for neurofibroma formation *in vitro* *Nf1^−/−^* SKPs were co-cultured with *Nf1^+/−^* DRGs, sciatic nerves, adipose and muscle tissues in an *in vitro* raft culture **A.** Rafts were harvested 4 months after setup for X-gal staining to trace the SKPs that are clustered within nerve tissues (red arrow) but not in muscle/adipose tissues (black arrow), indicating SKPs are neurotropic **B.** A DRG infiltrated by SKPs **C.** Histological analysis revealed that SKPs incorporated into nerve tissue **D.** Many areas within the *in vitro* raft culture show hyperplasia of LacZ-positive disorganized cells with wavy nuclei **E-G.** and these areas are positively stained with Schwann cell marker S100 **H.** In control experiments, nerve tissue did not show any sign of neoplasm **I-J.** Scale bar = 50 μm.

Primary neurofibroma tumor cells isolated from patients have a very limited proliferative capacity *in vitro* and undergo senescence within a short number of passages. Therefore, we sought to understand whether nerve environment can support neurofibroma development in the *in vitro* setting. To achieve this, we established a 3D skin raft culture system for neurofibroma formation *in vitro* with *Nf1^−/−^* SKPs and *Nf1^+/−^* DRGs and sciatic nerves in a collagen type I and dermal fibroblasts based raft culture system. In this particular setting, we also added *Nf1^+/−^* muscle and adipose tissue as non-nerve tissue controls. These skin rafts were cultured *in vitro* for up to 4 months prior to histological and molecular analysis (Figure 4A). Our whole mount observations revealed that SKPs are neurotropic and clustered with nerve tissues but not with control muscle or adipose tissues in culture (Figure 4B and 4C). Histologic analysis confirmed that SKPs infiltrated into nerve tissues (Figure 4D), and differentiate into Schwann cells *in vitro* as we have shown previously [27]. When we harvest these skin reconstructs for histological analysis, we found that these nerves/DRGs within the skin reconstructs exhibit the classic characteristics of human plexiform neurofibromas, being poorly circumscribed, composed primarily of spindle cells, and expressing the Schwann cell marker S100 (Figure 4E-4H). We also observed majority of cells within the neurofibromas in these skin reconstructs are also LacZ positive (Figure 4G), indicating that they originate from the *Nf1^−/−^* SKPs. In all experiments, control *Nf1^+/+^* SKPs or *Nf1^−/−^* SKPs alone did not give rise to neurofibroma (Figure 4I and 4J). These findings indicate that *Nf1*-deficient SKPs can give rise to *bona fide* neurofibromas in a skin raft culture system *in vitro*, contingent on their local microenvironment. Moreover, the essential role of peripheral nerves in the tumor microenvironment for neurofibroma genesis is underscored.

In human clinical scenario, mast cells infiltration is a characteristic of neurofibroma [28] and infiltrating *Nf1* heterozygous mutant bone marrow-derived mast cells play a critical role in driving neurofibroma development in a mouse model [10]. Given that our *in vitro* skin raft culture system lacks the hematopoietic components of the *in vivo* system; it is possible that in the physiological setting, neurofibromagenesis occurs stochastically and requires the contributions of all tumor microenvironmental factors (e.g., nerve, hematopoietic cells, *Nf1* heterozygosity in other cell types, fibroblasts, and endothelial cells). On the other hand, in the *in vitro* skin raft or *in vivo* nude mice transplant experimental settings, contributions from a large pool of *Nf1* homozygous tumor initiating cells and a supper physiologic level of nerve environment may override the otherwise requisite influences from other factors, such as mast cells or *Nf1* heterozygosity in other cells within the microenvironment, to induce neurofibroma development.

A tremendous gap exists between available culture and animal models and methods that can extract detailed information on cell-cell communication networks governing neurofibroma development. In addition, elucidating vital aspects of the interplay of extracellular factors in multi-population cellular systems is crucial for understanding neurofibroma pathophysiology but is exceedingly difficult to study *in vivo* or in traditional cell culture systems. Therefore, our 3D *in vitro* neurofibroma models provide an important alternative to both complex *in vivo* whole organism approaches, and 2D culture with its spatial constraints. Therefore, although our 3D *in vitro* neurofibroma model has limitations relative to existing animal models, including resource and time demands, translational potential and robustness, it provides the opportunities that allow us to elucidate the evolution of neurofibromagenesis and to elucidate the interactions between different cell types within a defined neurofibroma tumor microenvironment *in vitro* to delineate the pathogenesis as well as preclinical models for therapeutic testing.

In summary, our data indicate that genetically ablating *Nf1* in the cell of origin of neurofibroma is required but not sufficient for tumorigenesis, pointing to the essential role of the additional signals from the tumor microenvironment, including peripheral nerves. We speculate that neuron-derived growth factors, including but not limited to Nrg1 [23], may potentially act as exogenous drivers to sustain neurofibroma proliferation in a neuron-Schwann cell juxtacrine fashion. These are exciting results that offer an additional area of investigation for mechanisms vital to neurofibromagenesis. Our findings suggest this interaction between neoplastic Schwann cells and their surrounding nerves could be a potential therapeutic target for neurofibroma therapy.

## MATERIALS AND METHODS

### Mice

All animal care and use were approved by the Institutional Animal Care and Use Committee at University of Texas Southwestern Medical Center. *Nf1^flox^* and *P53^flox^* mouse was reported previously [17], Rosa26-LacZ and athymic nude mice were obtained from the Jackson Laboratory.

### Isolation and culture of SKPs

The isolation of SKPs was reported previously [29]. Briefly, dorsal skin was harvested from mouse neonatal pups (P0-P2). Adipose and muscle tissues on skin were carefully dissected out. The skin was rinsed three times in HBSS (Invitrogen) and then chopped into small pieces (2–3 mm) by sterilized equipment. To dissociate single cells from the tissue, skin was incubated with 0.5% collagenase I (Invitrogen) at 37°C for 1 hr with gentle shacking. To further dissociate the cells from tissues, the digested tissues were pipetted several times. Un-dissociated tissues were removed by 70 μm cell strainer. Cells in suspension were pelleted and then washed three times with serum-free DMEM/F12 media (Invitrogen). Skin cells were plated at a density of 20 cells/μl on ultra-low attachment plates (Corning) in SKP proliferation media [DMEM/F12 containing penicillin/streptomycin (0.1%); fungizone (40 μg/ml); B27 (without vitamin A), epidermal growth factor (20 ng/ml), and basic fibroblast growth factor (40 ng/ml; Sigma)]. SKPs form spheres by time of culture. SKPs were fed by fresh media every 3 - 4 days and passaged every 7 days.

### Generation of *Nf1^−/−^* and *Nf1^−/−^;P53^−/−^* SKPs

*Nf1^−/−^* SKPs were generated from *Nf1^flox/flox^;R26R* SKPs followed by transduction by adenovirus carrying Cre recombinase (Ad-CMV-Cre) as described previously [13]. An efficient Cre-mediated gene ablation was verified by *Nf1* genotyping and Xgal staining. *Nf1^flox/flox^;R26R* SKPs were also infected with Ad-CMV-GFP as *Nf1^+/+^* control. Likewise, *Nf1^−/−^;P53^−/−^* SKPs were generated from *Nf1^flox/flox^;P53^flox/flox^* SKPs by the same strategy.

### Allograft transplantation of SKPs into athymic nude mice

Athymic nude mice were anesthetized by intraperitoneal injection of 100 μl (per 25 g) of a mixture of ketamine (10 mg/ml) and xylazine (1 mg/ml) solution. For allograft transplantation, 1 × 10^6^ dissociated SKP single cells were resuspended in 40 μl of L15 medium (GIBCO) and injected into the subcutaneous tissue or sciatic nerve in nude mice. The sites for subcutaneous injection were marked by tattoo for future identification. Sciatic nerve is located above femur and the sciatic nerve implantation was performed described previously [13].

### Generation of *in vitro* engineered tissue rafts

The *in vitro* engineered tissue raft was generated as previously described with modifications [27]. Briefly, 24-mm tissue culture inserts for six-well plates (Transwell Permeable Supports, Corning) were coated with 60% of bovine collagen I (Organogenesis) in DMEM / 10% FBS as an acellular layer in the bottom followed by incubation at 37°C for 1 hr for solidification. The cellular layer was prepared by mixing of 1 × 10^6^ SKPs, 2 × 10^5^ human foreskin fibroblasts, and nerve tissues as described in DMEM with 10% FBS, 75% collagen I, 50 ng/ml heregulin (R&D Systems), and 5 μM forskolin (Sigma). Nerve tissues (DRG and sciatic nerve) were freshly isolated from adult *Nf1^+/−^* mice, dissect into small pieces, and then added into the cellular layer mixture. The cellular layer was layered on top of acellular layer and incubated at 37°C for 1 hr for solidification. The rafts were kept submerged in regularly refreshed DMEM medium with 10% FBS. Two to five months later, tissue rafts were harvested and subjected to for histological and immunohistochemical analysis.

### X-gal staining

To identify the *Nf1^−/−^* SKPs and cells differentiated from them, X-gal staining was performed to trace the cells with LacZ activity as a marker from Rosa26 reporter. Tissues were harvested and fixed in 4% paraformaldehyde in PBS for 1 hr, and then equilibrated in 30% sucrose in PBS for overnight at 4°C. Tissues were then rinsed in PBS three times and stained in X-gal staining solution at 30°C for overnight. The X-gal staining solution was freshly prepared by mixing following ingredients in PBS at their final concentrations: 1 mg/ml X-gal, 4 mmol/l potassium ferrocyanide, 4 mmol/l potassium ferricyanide, and 2 mmol/l magnesium chloride. X-gal stained tissues were fixed in 10% formalin for overnight.

### Histology and immunohistochemistry analysis

For histological analysis, tissue specimens (with or without prior X-gal staining) were fixed in 10% formalin for overnight and then subjected to paraffin embedding and tissue sectioning at 5-μm thickness. Hematoxylin and eosin (H&E) staining was performed on deparaffinized and rehydrated tissue sections with manufacturer's protocol (StatLab). For immunohistochemistry (IHC) analysis, tissue sections were deparaffinized, rehydrated, and then subjected to antigen retrieval. Tissues were blocked by 10% goat serum in PBS for 1 hr prior to incubation with the primary antibodies at 4°C for overnight. Biotinylated secondary antibodies were selected based on the origin of primary antibodies. Peroxidase-conjugated avidin was applied to amplify the signals. Final chromogenic reaction was performed by 3, 3′-diaminobenzidine (DAB) according to manufacturer's protocol (Vector Labs). Schwann cell markers S100 (Dako, Z0311) and GAP43 (Abcam, ab75810) are the primary antibodies used in this study.
